# Catalytic Modification of Porous Two-Dimensional Ni-MOFs on Portable Electrochemical Paper-Based Sensors for Glucose and Hydrogen Peroxide Detection

**DOI:** 10.3390/bios13050508

**Published:** 2023-04-28

**Authors:** Ya Yang, Wenhui Ji, Yutao Yin, Nanxiang Wang, Wanxia Wu, Wei Zhang, Siying Pei, Tianwei Liu, Chao Tao, Bing Zheng, Qiong Wu, Lin Li

**Affiliations:** 1 Key Laboratory of Flexible Electronics (KLOFE), Institute of Advanced Materials (IAM), Nanjing Tech University (NanjingTech), Nanjing 211800, China; iamyayang@njtech.edu.cn (Y.Y.); iamjwh@njtech.edu.cn (W.J.); ytyin@njtech.edu.cn (Y.Y.); wnx199703@163.com (N.W.); iamwxwu@njtech.edu.cn (W.W.); iamwzhang@njtech.edu.cn (W.Z.); iamsypei@njtech.edu.cn (S.P.); iamtwliu@njtech.edu.cn (T.L.); 2 The First Affiliated Hospital of Nanjing Medical University, Nanjing 210029, China; taochao@jsph.org.cn; 3 The Institute of Flexible Electronics (IFE, Future Technologies), Xiamen University, Xiamen 361005, China

**Keywords:** Ni-MOFs, paper-based sensors, electrochemical sensor, non-enzymatic catalysis, hydrogen peroxide, glucose

## Abstract

Rapid and accurate detection of changes in glucose (Glu) and hydrogen peroxide (H_2_O_2_) concentrations is essential for the predictive diagnosis of diseases. Electrochemical biosensors exhibiting high sensitivity, reliable selectivity, and rapid response provide an advantageous and promising solution. A porous two-dimensional conductive metal–organic framework (cMOF), Ni-HHTP (HHTP = 2,3,6,7,10,11-hexahydroxytriphenylene), was prepared by using a one-pot method. Subsequently, it was employed to construct enzyme-free paper-based electrochemical sensors by applying mass-producing screen-printing and inkjet-printing techniques. These sensors effectively determined Glu and H_2_O_2_ concentrations, achieving low limits of detection of 1.30 μM and 2.13 μM, and high sensitivities of 5573.21 μA μM^−1^ cm^−2^ and 179.85 μA μM^−1^ cm^−2^, respectively. More importantly, the Ni-HHTP-based electrochemical sensors showed an ability to analyze real biological samples by successfully distinguishing human serum from artificial sweat samples. This work provides a new perspective for the use of cMOFs in the field of enzyme-free electrochemical sensing, highlighting their potential for future applications in the design and development of new multifunctional and high-performance flexible electronic sensors.

## 1. Introduction

Owing to severe global environmental pollution, aging populations, and increasing sub-healthy populations, health problems have become a major public concern, making the early prevention and diagnosis of diseases crucial. To this end, the design and establishment of highly sensitive, specific, and rapid analysis methods for disease-related biomarkers are essential. Human body fluids (such as sweat, blood, tears, and saliva) contain a large amount of biochemical information that can reflect the health status of the human body [1]. For example, sweat or blood sodium and potassium ions reflect the amount of electrolytes in, or hydration level of, the human body [2]; sweat and blood cortisol can reveal the stress and mental state of humans [3]. Therefore, the detection of disease-related biomarkers in human body fluids can provide important references for the diagnosis and prevention of diseases. Hydrogen peroxide (H_2_O_2_) is a key reactive oxygen species that acts as a disease biomarker and a second messenger to maintain normal cellular function. The normal concentration of H_2_O_2_ in cells ranges from 0.001 to 0.1 μM [4,5,6,7]. When H_2_O_2_ concentration is high, it causes oxidative stress, which usually leads to various diseases, such as Parkinson’s disease and cancer [8,9,10,11]. H_2_O_2_ is also one of the products of oxidative enzymes, such as lactate oxidase; a catalyzing target; and can be used to indirectly detect the concentration of relevant biomarkers, such as lactate [9,10,12]. Diabetes is a metabolic disease that is mainly characterized by elevated glucose (Glu) concentrations [13]. The normal concentrations of Glu in blood and sweat are 4.9–6.9 mM and 0.23–0.38 mM, respectively [14]. When the concentration of Glu is extremely high, it may cause blindness, weight loss, kidney disease, neurological disorders, and cardiovascular disease in addition to diabetes [15,16,17,18,19]. Therefore, the accurate detection of H_2_O_2_ and Glu in human body fluids is essential for the diagnosis and prevention of related diseases [20].

Common detection methods for H_2_O_2_ and Glu include spectrophotometry, chemiluminescence, titrimetric analysis, colorimetry, and chromatography; however, these methods generally suffer from low sensitivity, cumbersome operation steps, long process durations, and high costs, limiting their wide application [21,22,23]. The recently developed electrochemical method has the advantages of high specificity and sensitivity for real-time monitoring; it is also inexpensive and portable and involves a simple operation procedure [24,25]. Traditional electrochemical Glu and H_2_O_2_ sensors are mainly based on glucose oxidase (GOX) and horseradish peroxidase (HRP), respectively; these enzymatic electrochemical sensors are often limited by their susceptibility to external environmental factors, such as temperature and light [26]. Therefore, in recent years, researchers have begun to focus on enzyme-free electrochemical sensors owing to their high sensitivity, excellent stability, and wide linear range [27]. Various biomimetic materials have been developed for the construction of enzyme-free sensors [28,29]. The design and preparation of H_2_O_2_ enzyme-mimetic materials can not only improve and expand the detection performance and application of H_2_O_2_ sensors but also enhance the detection performance of enzyme-based sensors that form H_2_O_2_ as a product. For example, lactate oxidase and GOX catalyze lactate and Glu, respectively, to produce H_2_O_2_, which can be further decomposed by H_2_O_2_ enzyme-mimetic materials to produce electrons [30]. Similarly, the design and preparation of Glu enzyme-free sensors are significant and facilitate practical application. Although numerous Glu enzyme-mimetic materials have been developed, their selectivities are still lower than that of GOX; therefore, the design and preparation of both enzyme and enzyme-free sensors for Glu are similarly critical.

Metal–organic frameworks (MOFs) are networks of porous crystals with organic linkers that act as “struts” and metal clusters as “joints” through coordination bonds or molecular interactions. These structures impart MOFs with both the flexibility of organic materials and the rigidity of inorganic materials [31,32,33]. High surface areas, tunable pores, and ordered crystal structures make MOFs potential candidates as enzyme-mimetic materials [31,34]. However, the poor electrical conductivity of MOFs limits their application in biosensing. One typical solution is to combine MOFs with other active materials, such as carbon nanotubes or metal particles, to improve their electrochemical properties [35]. Among such active materials, transition metals are of considerable interest because of their low cost, excellent conductivity, high catalytic activity, and ease of preparation [36]. Conductive MOFs (cMOFs) possess the advantage of having highly porous structures [37], abundant catalytic active sites [38], and intrinsic electrical conductivity [39], enabling them to overcome the shortcomings of MOFs and thus become an ideal multifunctional material for electrochemical sensing applications [40,41,42]. In general, the catalytic sites of cMOFs are mainly their metal centers, indicating that the metal centers directly determine the intrinsic electrocatalytic capacity of cMOFs [43,44].

Whitesides et al. first introduced the concept of paper-based microfluidic chips in 2007 and used optical and electrochemical methods to develop a variety of paper-based sensors for various applications, such as clinical diagnosis and environmental monitoring [45,46,47,48,49]. Paper-based sensors have many advantages, including the ability to self-actuate, good three-dimensional fiber structure, suitable biodegradability and biocompatibility, simple preparation and modification processes, and low cost [50]. For example, an inexpensive and highly combined wearable paper-based sensor was designed by Li et al. for the precise analysis of Glu and lactate in human sweat during exercise [51]; Jiao et al. developed a novel three-dimensional vertical flow paper device and combined it with a sandwich-type fluorescence assay for the simultaneous detection of multiple cancer biomarkers [52]. In addition, several researchers have modified the surfaces of paper-based sensors with nanomaterials or chemical treatments, which substantially improved their physical and chemical properties [53,54,55]. Therefore, paper-based sensors have a broad development potential and good research prospects.

In this study, a nanosized porous two-dimensional MOF, Ni-HHTP (HHTP = 2,3,6,7,10,11-hexahydroxytriphenylene), was prepared by using a one-pot method. Then, a contamination-free, flexible, and mass-producible paper-based electrochemical sensor was fabricated by applying screen and inkjet printing techniques. Finally, Ni-HHTP was modified onto the surface of the paper-based electrode by drop coating; the resulting device achieved enzyme-free, rapid, and highly sensitive detection of Glu and H_2_O_2_. We verified that Ni-HHTP had a porous structure and exhibited excellent electrical conductivity and electrocatalytic properties, using H_2_O_2_ and Glu as detection models. Furthermore, the H_2_O_2_ and Glu sensors achieved low limits of detection (LODs; 2.13 μM and 1.30 μM, respectively), high sensitivity (179.85 μA μM^−1^ cm^−2^ and 5573.21 μA μM^−1^ cm^−2^, respectively), and excellent selectivity, which can be used to detect H_2_O_2_ in human serum and Glu in artificial sweat. This study presents a novel role for MOFs in the field of non-enzymatic electrochemical sensing and highlights their extensive potential applications in the design and development of new multifunctional and high-performance flexible electronic sensors.

## 2. Materials and Methods

### 2.1. Reagents and Materials

Nickel acetate tetrahydrate (Ni(Ac)_2_·4H_2_O) was purchased from Sinopharm (Beijing, China). Moreover, 2,3,6,7,10,11-hexahydroxytriphenyl (HHTP) was acquired from Shanghai Tengqian Co., Ltd. (Shanghai, China). Potassium ferricyanide (K_3_Fe[(CN)_6_]), sodium hydroxide (NaOH), sodium chloride (NaCl), calcium chloride (CaCl_2_), potassium chloride (KCl), potassium ferricyanide (K_4_[Fe(CN)_6_]), and hydrogen peroxide (H_2_O_2_, 30%) were obtained from Shanghai Aladdin Biochemical Technology Co., Ltd. (Shanghai, China). Dopamine, urea, and L-cysteine were acquired from Nanjing Beyotime Biotechnology Co., Ltd. (Nanjing, China). Ascorbic acid and glucose were purchased from Shanghai Yuanye Biotechnology Co., Ltd. (Shanghai, China). Lactic acid was purchased from NanJing WanQing Chemical Glassware Instrument Co., Ltd. (Nanjing, China). Perfluorosulfonic acid resin (Nafion, 5%) was acquired from Shanghai Adamax Reagent Co., Ltd. (Shanghai, China). Anhydrous ethanol (EtOH) was acquired from Nanjing Evening Chemical Glass Instrument Co., Ltd. (Nanjing, China). Conductive carbon pulp and conductive silver pulp were purchased from Shanghai Julong Electronic Technology Co., Ltd. (Shanghai, China) Whatman^®^ Grade 5 qualitative filter paper was acquired from GE Healthcare Worldwide (Shanghai, China). Deionized water (18.2 MΩ cm) was obtained from a Milli-Q water purification system. Human serum was provided by the Nanjing Maternal and Child Health Hospital. Artificial sweat was prepared by dissolving 0.05 g urea, 0.25 g NaCl, and 47 μL 0.01 M lactic acid in 50 mL ultrapure water.

### 2.2. Material Characterization and Electrochemical Instruments

X-ray diffraction (XRD; Bruker, Beijing, China), field-emission scanning electron microscopy (SEM; Dayu Keyi, Beijing, China), and transmission electron microscopy (TEM; Tianmei, Beijing, China) were used to characterize the structures of the crystals and the morphological features of the materials. Fourier transform infrared (FT-IR) spectroscopy (RuiJie, Tianjin, China), energy-dispersive X-ray spectroscopy (EDS; Mahwah, Nanjing, China), Brunauer–Emmett–Teller (BET) surface area (Gold APP, Beijing, China) and pore size distribution data were also used for material characterization. Chronoamperometry (CA), differential pulse voltammetry (DPV), electrochemical impedance spectroscopy (EIS), and cyclic voltammetry (CV) were conducted using the Palmsens4 electrochemical workstation (RED MATRIX, Guangzhou, China). The Xerox Color Qube 8570 wax printer was purchased from Xerox (Norwalk, CT, USA).

### 2.3. Synthesis and Preparation of Ni-HHTP

**Scheme 1:**
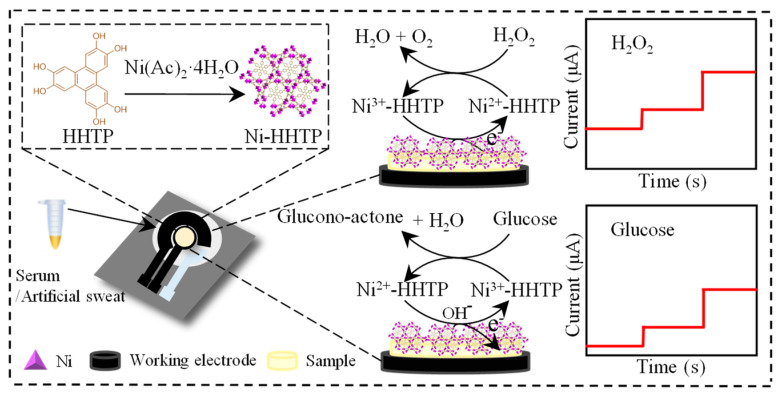
Schematic diagram of Ni-HHTP synthesis and detection mechanism of paper-based sensor based on Ni-HHTP.

First, 25 mg of Ni(Ac)_2_·4H_2_O was dissolved in 5 mL of ultrapure water in a glass vial, which was then placed in an ultrasonicator to fully disperse it; 1 mL of the dispersion was taken as solution A. Similarly, 35 mg of HHTP was dissolved in 30 mL of ultrapure water in a glass vial, which was then placed in the ultrasonicator to fully disperse the HTTP; 6 mL of the dispersion was taken as solution B. Then, 1 mL of solution A was mixed with 6 mL of solution B in a 50 mL glass vial, following which 13 mL of ultrapure water was added to the mixture. The glass vial was placed in an oven and heated at 85 °C for 12 h, resulting in the formation of black lumps. These were allowed to cool naturally to room temperature, and then washed three times by employing centrifugation with 1 mL of ultrapure water and EtOH, and then dried overnight at room temperature under vacuum to obtain the final black powdered crystals (Scheme 1).

### 2.4. Fabrication of Paper-Based Electrochemical Sensors

A schematic diagram of the Ni-HHTP/screen-printed carbon electrode (SPCE) fabrication process is shown in Figure S1. The detection zone was divided into a hydrophilic zone in the middle and a hydrophobic zone in the outer layer. The hydrophobic zone was daubed with wax using the Xerox Color Qube 8570 wax printer and then dried at 120 °C for 2.5 min to promote wax penetration into the paper. The SPCE, a three-electrode system, was then fabricated via screen printing. Carbon paste was applied as the counter electrode (CE) and the working electrode (WE); after screen printing the WE and CE, the printed electrode was dried at 60 °C for 30 min. Silver paste was then printed and dried at 40 °C for 20 min to prepare the reference electrode (RE). The printed paper was cut into 3 cm × 2 cm electrode arrays, and double-sided tape was applied to their backs to prevent liquid leakage.

### 2.5. Modification of Paper-Based Electrochemical Sensors

The WE was functionalized with Ni-HHTP using the drop-coating method. First, 1.4 mg of Ni-HHTP was fully dissolved in 200 μL EtOH and 200 μL ultrapure water to obtain a 3.5 mg/mL Ni-HHTP solution. Second, 20 μL of the 3.5 mg/mL Ni-HHTP solution and 2 μL of 0.1% Nafion solution were mixed, following which they were vortexed for 15 min and sonicated for 30 min. Finally, 2 μL of the manufactured Ni-HHTP mixture was applied uniformly on the WE using a microsampler and dried naturally at room temperature.

## 3. Results and Discussion

### 3.1. Material Characterization

**Figure 1:**
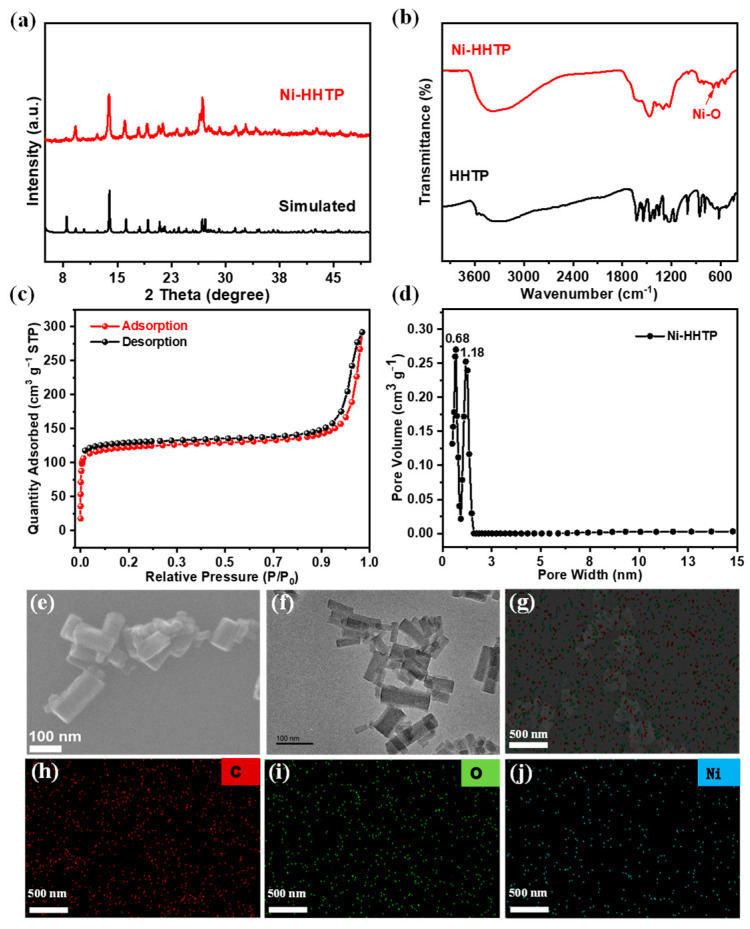
Material characterization. (**a**) XRD patterns of Ni-HHTP; (**b**) FTIR spectra of Ni-HHTP and HHTP; (**c**,**d**) BET surface area and pore size distribution of Ni-HHTP; (**e**,**f**) SEM and TEM images of Ni-HHTP; (**g**) EDS layered images of Ni-HHTP; (**h**–**j**) EDS elemental mapping images of Ni-HHTP.

The characteristic structures of the crystals were verified by performing powder XRD. The XRD pattern of Ni-HHTP showed diffraction peaks at 2θ = 4.5°, 9.3°, 13.8°, and 27.0°, which corresponded to the (100), (020), (002), and (111) crystal planes, respectively [40] (Figure 1a). The FT-IR spectra of HHTP and Ni-HHTP are shown in Figure 1b. For HHTP, peaks were observed at 3300–3600 cm^−1^, indicating the presence of −OH; these peaks disappeared in the pattern for Ni-HHTP owing to the coordination of O in the hydroxyl group to Ni. The peaks at 1632 cm^−1^ and 1454 cm^−1^ were ascribed to the asymmetric vibration of O=C−O, whereas those at 1218 cm^−1^ and 1308 cm^−1^ represented C−O. A new peak appeared at 806 cm^−1^, indicating the formation of the Ni−O bonds. The BET-specific surface area was 484.9 m^2^ g^−1^. Ni-HHTP exhibited a typical reversible type I adsorption isotherm (Figure 1c). The average pore size calculated by using the density function theory (DFT) model was approximately 1.18 nm (Figure 1d), which is consistent with the crystallographic structure of the material [56].

The composite Ni-HHTP material was also characterized by using SEM and TEM to further explore its structural and morphological features. As revealed in Figure 1e,f, Ni-HHTP has a relatively regular columnar structure with a small size of approximately 100 nm in diameter. The EDS layered image of Ni-HHTP further characterized the microporous structure of the Ni-HHTP surface (Figure 1g), whereas its corresponding elemental mapping patterns proved the presence of elemental Ni and its complete overlap with the distribution of elemental C and O (Figure 1h-j). This led us to conclude that the metal Ni and the organic ligand HHTP underwent reciprocal coordination to form a two-dimensional layered crystal structure.

### 3.2. Optimization of Paper-Based Electrochemical Sensor

Among all the transition metals, Ni metal usually exhibits a large number of excellent properties as an electrochemical sensor, including high sensitivity, low detection limits, and favorable stability, as well as a wider linear range compared to other metals [32,57,58]. The key ligand, HHTP, greatly contributes to the charge leaving domain owing to its good orbital overlap with the metal center, which enhances the holistic electrical conductivity of the material [59,60]. To enhance the detection performance of the sensor, the test conditions were optimized. First, different excitation potentials of 0.2, 0.3, 0.4, and 0.45 V were tested using the chronoamperometry (CA) method, and 0.4 V was chosen as the definitive potential (Figure S2c). Second, the catalytic effect of the MOF and Nafion at different ratios (3.5 mg/mL MOF: 0.1% Nafion = 1:1, 2:1, 5:1, 10:1, and 25:1) (Figure S2a) were compared, and the results indicated that the optimal ratio was 10:1. The effect of the number of activation cycles (40, 60, 80, and 100 cycles) on the sensor was also evaluated, and the results indicated that the oxidation and reduction currents gradually increased with an increasing number of activation cycles, and reach the equilibrium state was reached after 80 activation cycles (Figure S2b). In addition, in order to ensure that the amount of H_2_O_2_ molecules entering the MOF pores and being adsorbed was sufficient, the pre-enrichment time of the analyte at the electrode was optimized by comparing the current change before and after different enrichment times, and was found to be 120 s (Figure S2d). Generally, the solution contains dissolved oxygen, and its presence can sometimes hinder the redox process. Therefore, the sensor was tested to investigate whether argon protection by purging the dissolved oxygen in the solution was necessary; the results showed that argon sealing protection was dispensable to the test system (Figure S2e). Different processes by which nickel changed during the pre-activation process are shown in Figure S2f. As shown in Figure S6a, by testing the CA of Ni-HHTP/SPCE at the excitation potential of 0.4, 0.5, 0.6, and 0.7 V, and comparing the slope of the fitting curve. It was concluded that the best excitation potential of Glu tested by CA is 0.5 V. Because the cyclic voltammetry (CV) oxidation peak current of Glu was not obvious with the increase of concentration, we used a more sensitive differential pulse voltammetry (DPV) to detect Glu. It can be seen that the oxidation peak current rose gradually with growth concentrations (Figure S6b,c).

### 3.3. Electrochemical Responses of the Sensors to H_2_O_2_ and Glu

**Figure 2:**
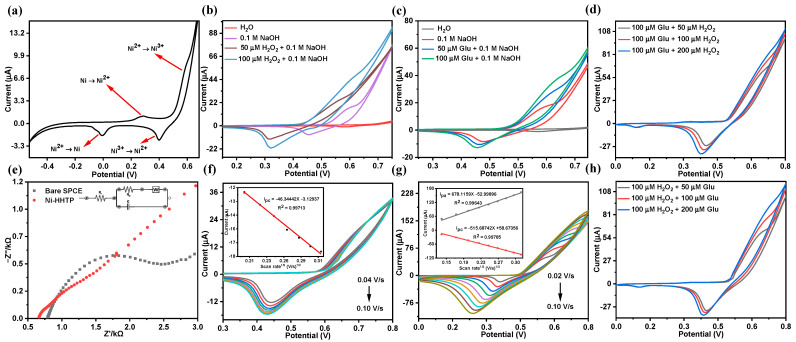
Electrochemical responses of the sensors to H_2_O_2_ and Glu. (**a**) Electron transfer reactions during Ni-HHTP/SPCE activation. (**b**) CV of Ni-HHTP/SPCE in H_2_O, 0.1 M NaOH, 0.1 M NaOH containing 50 μM H_2_O_2_, and 0.1 M NaOH containing 100 μM H_2_O_2_. (**c**) CV of Ni-HHTP/SPCE in H_2_O, 0.1 M NaOH, 0.1 M NaOH containing 50 μM Glu, and 0.1 M NaOH containing 100 μM Glu. (**d**) CV of Ni-HHTP/SPCE in 100 μM Glu containing 0.1 M NaOH mixed with different concentrations of H_2_O_2_. (**e**) EIS plot and fitting circuit of Ni-HHTP/SPCE. (**f**) CV of Ni-HHTP/SPCE at different scan rates (0.04, 0.05, 0.06, 0.07, 0.08, 0.09 and 0.10 V/s) in 50 μM H_2_O_2_ (inset shows the fitted curves of I_pc_ versus the V^1/2^). (**g**) CV of Ni-HHTP/SPCE at different scan rates (0.02, 0.03, 0.04, 0.05, 0.06, 0.07, 0.08, 0.09 and 0.10 V/s) in 100 μM Glu (insets show the fitted curves of I_pa_ versus the V^1/2^). (**h**) CV of Ni-HHTP/SPCE in 100 μM H_2_O_2_ containing 0.1 M NaOH mixed with different concentrations of Glu.

A two-dimensional porous Ni-HHTP material with high catalytic activity was used to prepare an electrochemical paper-based sensor for detecting H_2_O_2_ and Glu. To increase the electron transport efficiency and catalytic performance of Ni-HHTP, the sensor was pre-activated in 0.1 M NaOH for 80 cycles to remove the trapped solvent inside the Ni-HHTP pore channel. The interconversion between Ni^2+^ and Ni^3+^ can be achieved in alkaline solution [37,40,61]. We also performed a control experiment, which was pe-formed in PBS solution, and found that it did not produce the typical redox signals of Ni^2+^ and Ni^3+^ (Figure S5). The oxidation peak represents the process of Ni and Ni^2+^ losing electrons to Ni^2+^ and Ni^3+^, respectively, while the reduction peak signifies the process of Ni^2+^ and Ni^3+^ gaining electrons from Ni and Ni^2+^, respectively (Figure 2a). In addition, the conductivity of Ni-HHTP was analyzed using EIS (Figure 2e), and it was found that when the bare electrode was modified with Ni-HHTP, its charge transfer resistance (R_ct_) decreased significantly from 1420 Ω to 660.6 Ω, indicating that Ni-HHTP possessed good conductivity. The reaction processes on the electrode surface were also investigated. Next, we investigated the relationship between the current and the scan rate for the Ni-HHTP/SPCE electrode (Figure S3a,b). The oxidation peaks shifted toward a more positive potential with increasing scan rate, and a linear relationship (R^2^ = 0.9912) was observed between the oxidation peak current (I_pa_) and the square root of the scan rate (V^1/2^), indicating that the electrochemical reaction of the potassium ferricyanide system in Ni-HHTP/SPCE was a typical diffusion-controlled process.

We then evaluated the H_2_O_2_ and Glu detection performances of the prepared sensors. Ni-HHTP/SPCE generated weak currents in ultrapure water and NaOH and exhibited relatively positive potentials. In comparison, the current generated by Ni-HHTP/SPCE for H_2_O_2_ increased significantly, and the peak shifted toward a negative potential. Furthermore, as the H_2_O_2_ concentration increased, the current increased further, indicating that Ni-HHTP was effective in catalyzing H_2_O_2_ (Figure 2b). Figure 2f shows the CV of Ni-HHTP/SPCE in H_2_O_2_ at different scan rates. When the scan rate gradually increased, the current increased as well, and the peak moved toward a relatively negative potential. Similar to the observation regarding the oxidation peak, a linear relationship was observed between the reduction peak current (I_pc_) and the V^1/2^ (R^2^ = 0.9971), indicating a diffusion-controlled process of H_2_O_2_ reduction on the Ni-HHTP/SPCE, which means that quantitative analysis can be conducted. The CV curves in Figure 2c show that the current increased gradually with increasing Glu concentration, further indicating that Ni-HHTP catalyzed the oxidation of Glu. The reaction mechanism of Glu and H_2_O_2_ based on Ni-HHTP/SPCE is as follows [62,63,64]:

(1) $$Ni^{2+}-\mathrm{MOF}\to Ni^{3+}-\mathrm{MOF}+e^{-}$$

(2) $$Ni^{3+}-\mathrm{MOF}+OH^{-}+glucose\to Ni^{2+}-\mathrm{MOF}+glucolactone+H_{2}O$$

(3) $$Ni^{2+}-\mathrm{MOF}+H_{2}O_{2}\to Ni^{3+}-\mathrm{MOF}+H_{2}O+O_{2}+e^{-}$$

The CV curves of Ni-HHTP/SPCE for Glu at different scan rates in Figure 2g exhibited a similar trend as well. The currents increased gradually as the scan rate increased, and the peak shifted toward a relatively negative potential. Further analysis revealed a linear relationship between the I_pa_ and the V^1/2^ (R^2^ = 0.9964), implying that Glu undergone a diffusion-controlled oxidation reaction on the Ni-HHTP/SPCE. In addition, we fixed the concentration of Glu and added different concentrations of H_2_O_2_ to it, and discovered that the I_pc_ increased gradually with increasing H_2_O_2_ concentration; however, the I_pa_ did not change significantly, indicating that the reduction reaction of H_2_O_2_ was catalyzed by Ni-HHTP (Figure 2d). Similarly, we fixed the H_2_O_2_ concentration (Figure 2h). The results showed that while the I_pa_ increased gradually with the increasing Glu concentration, its value did not change significantly; this proved that Glu underwent oxidation, which further confirmed that the two-dimensional conductive Ni-HHTP composite can be used for the common detection of dual biomarkers.

**Figure 3:**
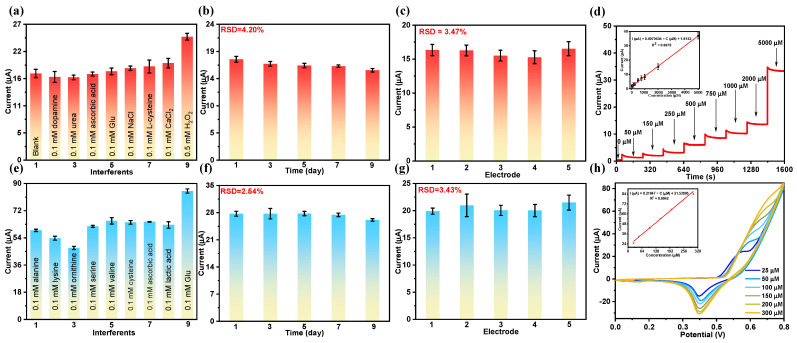
Selectivity, stability, repeatability, and linearity of the paper-based electrochemical sensors. (**a**) Selectivity of Ni-HHTP/SPCE for H_2_O_2_ detection (*n* = 9). (**b**) Changes in current generated by Ni-HHTP/SPCE in 0.1 M NaOH containing 50 μM H_2_O_2_ over 9 days. (**c**) Repeatability of Ni-HHTP/SPCE sensing in 0.1 M NaOH containing 50 μM H_2_O_2_ (*n* = 5). (**d**) CA of Ni-HHTP/SPCE at 0.4 V excitation potential for detection of different concentrations of H_2_O_2_ (inset shows the corresponding calibration curve of current versus H_2_O_2_ concentrations ranging from 0 to 5000 μM). (**e**) Selectivity of Ni-HHTP/SPCE Glu detection (*n* = 9). (**f**) Change in current generated by Ni-HHTP/SPCE in 0.1 M NaOH containing 100 μM Glu over 9 days. (**g**) Repeatability of Ni-HHTP/SPCE in 0.1 M NaOH containing 100 μM Glu (*n* = 5). (**h**) CV of Ni-HHTP/SPCE at different concentrations of Glu. Linear fitting curve of H_2_O_2_ concentration vs. current (inset shows the corresponding calibration curve of current versus Glu concentration).

The selectivity, stability, and repeatability of the prepared sensors were also evaluated. Among these, selectivity is of particular importance for non-enzymatic sensors, as they are often utilized to detect analytes in complex samples. Different interferents were selected. The plots in Figure 3a,e show that although Ni-HHTP/SPCE responded to the interferents, the response currents to H_2_O_2_ and Glu were the highest, evidencing that the prepared sensor possessed good selectivity. Moreover, the sensor exhibited reliable stability, as it could detect H_2_O_2_ and Glu even after 9 days of storage, with relative standard deviations (RSDs) of 2.54% for H_2_O_2_ and 4.20% for Glu (Figure 3b,f). Additionally, the RSDs became 3.47% and 3.43% when the tests were repeated (Figure 3c,g). In order to accurately quantify H_2_O_2_, CA was used to measure various H_2_O_2_ and Glu concentrations. As shown in Figure 3d,h, with the increment of concentration, the current increased gradually. The LOD of H_2_O_2_ and Glu reached 2.13 μM and 1.30 μM, respectively, according to the equation S1. The electrochemical surface area was calculated using the Randles–Sevcik equation. The result showed that the electrochemical surface area of Ni-HHTP/SPCE was 3.92 × 10^−5^ cm^−2^. The sensitivities of H_2_O_2_ and Glu were calculated as 179.85 μA μM^−1^ cm^−2^ and 5573.21 μA μM^−1^ cm^−2^ by the quotient between the slope of the fitting curve and the electrochemical surface area (Figure 3d,h) [65]. Compared with other MOF-based sensors (Tables S3 and S4), this sensor exhibits relatively higher sensitivity and lower LOD.

### 3.4. Application of Paper-Based Electrochemical Sensors

Two different biofluids (human serum and artificial sweat) were employed as application models to validate the performance of the Ni-HHTP/SPCE sensor for practical applications. Since the sensitivity of the Ni-HHTP/SPCE sensor is correlated with the NaOH concentration, in this trial, 10 μL of 100-fold-diluted human serum and 140 μL of 0.1 M NaOH were mixed well as the sample for the assay, and then added to the sensor chamber dropwise [41]. Note that the addition of the serum may dilute the NaOH concentration, which may affect the sensitivity of the sensor, as the sensitivity of the Ni-HHTP/SPCE sensor depends on the NaOH concentration [40]. Therefore, we first established a calibration standard curve for the Ni-HHTP/SPCE sensor in human serum using the standard addition method (Figure S4a), and then determined the recovery of 50 μM and 100 μM H_2_O_2_ from the human serum. The recoveries were in the range of 96.41–113.41%, with RSDs in the range of 1.91–3.03%, as shown in Table 1. The concentration of Glu in the artificial sweat was also detected using the standard addition method; the different concentrations of Glu in artificial sweat were calculated by using the linear relationship in Figure S4b. The recoveries ranged from 94.45–110.35%, with RSD values of 4.24% to 4.35% (Table 2). These results further confirmed the high reliability of the paper-based electrochemical sensor.

## 4. Conclusions

We designed and prepared a flexible and stable Ni-HHTP/SPCE sensor that combines the two-dimensional porosity, high conductivity, and catalytic properties of Ni-HHTP with the advantages of paper-based sensors via a one-pot method. The sensor detected H_2_O_2_ and Glu with high sensitivity and low detection limits. It also exhibited good selectivity for the target and showed excellent stability and reproducibility in multiple sets of sensors for up to 9 days. This study presents novel ideas for the use of cMOFs in non-enzymatic electrochemical sensing and highlights their widespread potential application in the design and development of new multifunctional and high-performance flexible electronic sensors.

## Figures and Tables

**Table 1:** H_2_O_2_ spiked recovery in human serum.

Count	Spiked (μM)	Detected (μM)	Recovery (%)	RSD (%)
1	50	48.20	96.41	3.03
2		53.48	106.96	
3		56.71	113.41	
4	100	102.40	102.40	1.91
5		107.21	107.21	
6		109.74	109.74	

**Table 2:** Glucose spiked recovery in artificial sweat.

Count	Spiked (μM)	Detected (μM)	Recovery (%)	RSD (%)
1	50	53.63	107.26	4.24
2		48.90	97.80	
3		47.22	94.45	
4	100	110.35	110.35	4.35
5		102.62	102.62	
6		99.07	99.07	

## Data Availability

Not applicable.

## Supplementary Materials

The following supporting information can be downloaded at: https://www.mdpi.com/article/10.3390/bios13050508/s1, Figure S1: Schematic diagram of the preparation of Ni-HHTP/SPCE paper-based electrochemical sensor; Figure S2: Condition optimization; Figure S3: (a) CV of Ni-HHTP/SPCE in a 1:1:1 mixture of 5 mM K_3_[Fe(CN)_6_], 5 mM K_4_[Fe(CN)_6_], and 0.1 M KCl at different scan rates (0.02, 0.04, 0.05, 0.07, and 0.09 V/s). (b) Linear fitting curve of oxidation peak current versus square root of scan rate. (c) CV of bare SPCE in H_2_O, 0.1 M NaOH, 0.1 M NaOH containing 50 μM H_2_O_2_, and 0.1 M NaOH containing 100 μM H_2_O_2_. (d) CV of bare SPCE in H_2_O, 0.1 M NaOH, 0.1 M NaOH containing 50 μM Glu, and 0.1 M NaOH containing 100 μM Glu; Figure S4: (a) CV of Ni-HHTP/SPCE in human serum (140 μL of 0.1 M NaOH with 10 μL of human serum) containing different concentrations of H_2_O_2_ (inset shows the fitted curve of the reduction peak current versus concentration). (b) CV of Ni-HHTP/SPCE in artificial sweat (140 μL of 0.1 M NaOH with 10 μL of artificial sweat) containing different concentrations of Glu (inset shows the fitted curve of the oxidation peak current versus concentration); Figure S5: PBS, PBS, and NaOH mixture control group; Figure S6: (a) The slope of Ni-HHTP/SPCE curve fitted by CA at the excitation potential of 0.4V, 0.5V, 0.6V, and 0.7V. (0.1M NaOH containing 50 μM Glu, 150 μM Glu, and 300 μM Glu). (b) CA of Ni-HHTP/SPCE at 0.5 V excitation potential for detecting different concentrations of Glu (inset shows the corresponding calibration curve of current versus Glu concentrations ranging from 0 to 200 μM). (c) DPV of Ni-HHTP/SPCE in 0.1 M NaOH containing 0 μM Glu, 100 μM Glu, 150 μM Glu, and 300 μM Glu, respectively; Table S1: Detection of blank sample (H_2_O_2_ sensor); Table S2: Detection of blank sample (Glucose sensor); Table S3: Comparison of MOF-based nonenzymatic glucose sensor; Table S4: Comparison of different nonenzymatic electrochemical H_2_O_2_ sensors. References [34,63,66,67,68,69,70,71,72,73,74] are cited in the Supplementary Materials.
