# Supplementary material for: Catalytic Modification of Porous Two-Dimensional Ni-MOFs on Portable Electrochemical Paper-Based Sensors for Glucose and Hydrogen Peroxide Detection
Source: Biosensors (Basel). 2023 Apr 28;13(5):508. doi: 10.3390/bios13050508 (PMC10216242; doi:10.3390/bios13050508)
Supplement: Supplementary file 1 [file biosensors-13-00508-s001.zip › biosensors-2306633-supplementary.pdf]

Supporting information

Catalytic Modification of Porous Two-Dimensional Ni-MOFs on Portable Electrochemical Paper-Based Sensors for Glucose and Hydrogen Peroxide Detection

Ya Yang [1,†], Wenhui Ji [1,†], Yutao Yin [1], Nanxiang Wang [1], Wanxia Wu [1], Wei Zhang [1], Siying Pei [1], Tianwei Liu [1], Chao Tao [2], Bing Zheng [1,*], Qiong Wu [1,*] and Lin Li [1,3,*]

[1] Key Laboratory of Flexible Electronics (KLOFE) & Institute of Advanced Materials (IAM), Nanjing Tech University (NanjingTech), Nanjing 211800, China; iamyayang@njtech.edu.cn (Y.Y.); iamjwh@njtech.edu.cn (W.J.); ytyin@njtech.edu.cn (Y.Y.); wnx199703@163.com (N.W.); iamwxwu@njtech.edu.cn (W.W.); iamwzhang@njtech.edu.cn (W.Z.); iamsypei@njtech.edu.cn (S.P.); iamtwliu@njtech.edu.cn (T.L.)
[2] The First Affiliated Hospital of Nanjing Medical University, Nanjing 210029, China; taochao@jsph.org.cn
[3] The Institute of Flexible Electronics (IFE, Future Technologies), Xiamen University, Xiamen 361005, China
* Correspondence: iambzheng@njtech.edu.cn (B.Z.); iamqwu@njtech.edu.cn (Q.W.); iamlli@njtech.edu.cn (L.L.)
† These authors contributed equally to this work.

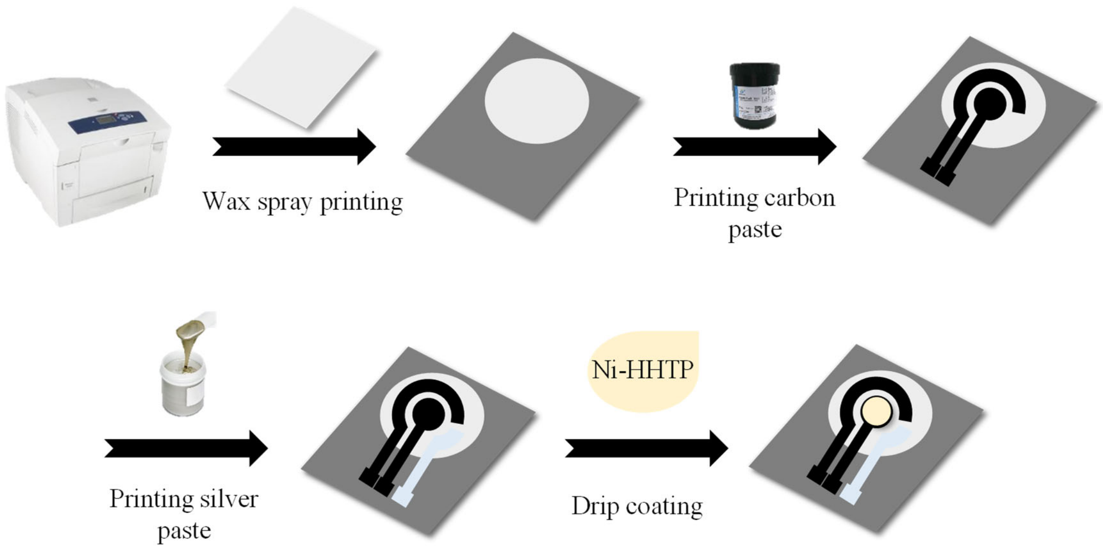

Figure S1. Schematic diagram of the preparation of Ni-HHTP/SPCE paper-based electrochemical sensor.

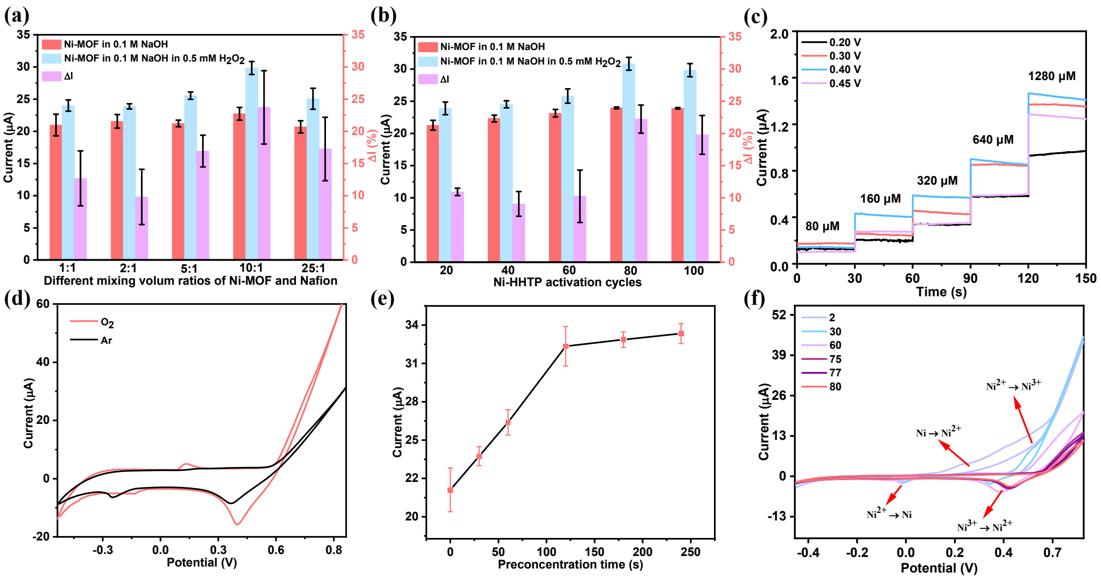

Figure S2. Condition optimization. (**a**) CV peak current of Ni-HHTP with 0.1% Nafion after modifying the electrode with different volume ratios in 0.1 M NaOH, 0.1 M NaOH containing 0.5 mM H_2O_2 (1:1, 2:1, 5:1, 10:1, 25:1). **(b)** CV peak current of Ni-HHTP/SPCE in 0.1 M NaOH, 0.1 M NaOH containing 0.5 mM H_2O_2 after different number of pre-activation cycles (20, 40, 60, 80, 100 cycles). **(c)** Chronoamperometry of Ni-HHTP /SPCE at excitation potential of 0.2, 0.3, 0.4 and 0.45 V, respectively (0.1 M NaOH containing 80 μM H_2O_2, 160 μM H_2O_2, 320 μM H_2O_2, 640 μM H_2O_2, 1280 μM H_2O_2). **(d)** CV of Ni-HHTP/SPCE in 0.1 M NaOH containing 0.5 mM H_2O_2 after 0, 30, 60, 120, 180 and 240 s of enrichment, respectively. **(e)** CV of Ni-HHTP /SPCE in 0.1 M NaOH containing 0.5 mM H_2O_2 with and without Ar environment. **(f)** CV of Ni-HHTP /SPCE at the 2nd, 30th, 60th, 75th, 77th and 80th cycles of the pre-activation.

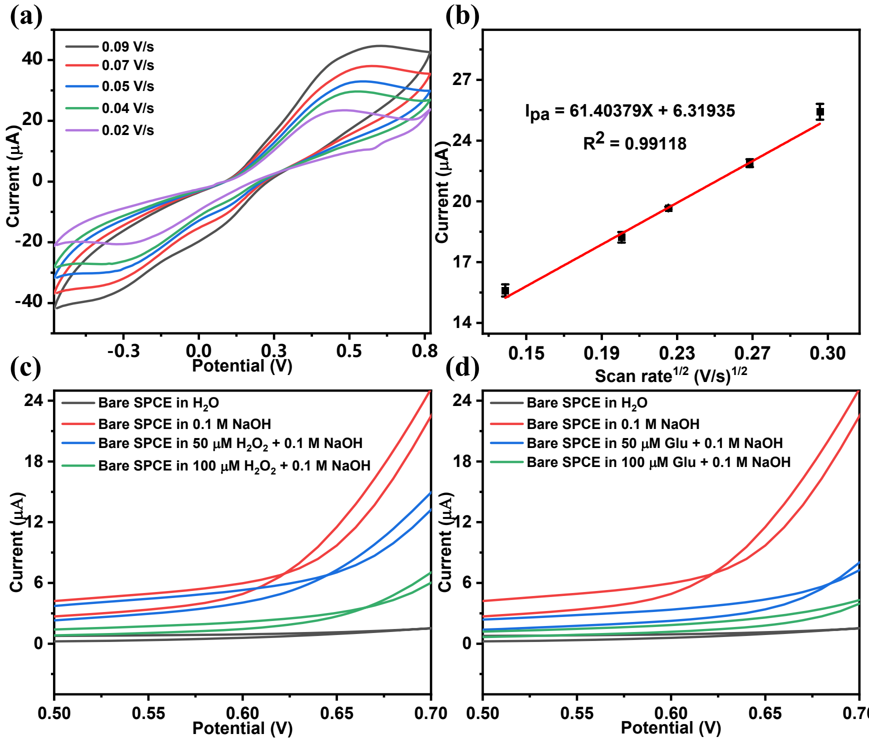

Figure S3. (a) CV of Ni-HHTP/SPCE in a 1:1:1 mixture of 5 mM $K_3Fe(CN)_6$, 5 mM $K_4[Fe(CN)_6]$, and 0.1 M KCl at different scan rates (0.02, 0.04, 0.05, 0.07, and 0.09 V/s). **(b)** Linear fitting curve of oxidation peak current versus square root of scan rate. **(c)** CV of bare SPCE in H_2O, 0.1 M NaOH, 0.1 M NaOH containing 50 μM H_2O_2, and 0.1 M NaOH containing 100 μM H_2O_2. **(d)** CV of bare SPCE in H_2O, 0.1 M NaOH, 0.1 M NaOH containing 50 μM Glu, and 0.1 M NaOH containing 100 μM Glu.

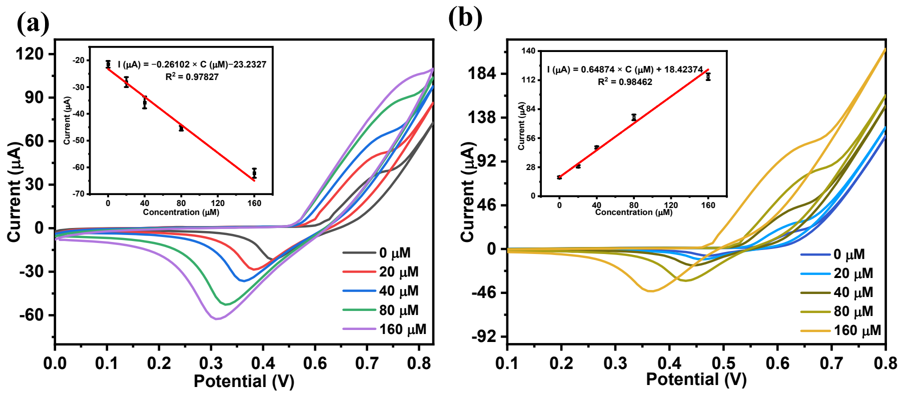

Figure S4. (a) CV of Ni-HHTP/SPCE in human serum (140 μL of 0.1 M NaOH with 10 μL of human serum) containing different concentrations of H_2O_2 (inset shows the fitted curve of the reduction peak current versus concentration). **(b)** CV of Ni-HHTP/SPCE in artificial sweat (140 μL of 0.1 M NaOH with 10 μL of artificial sweat) containing different concentrations of Glu (inset shows the fitted curve of the oxidation peak current versus concentration).

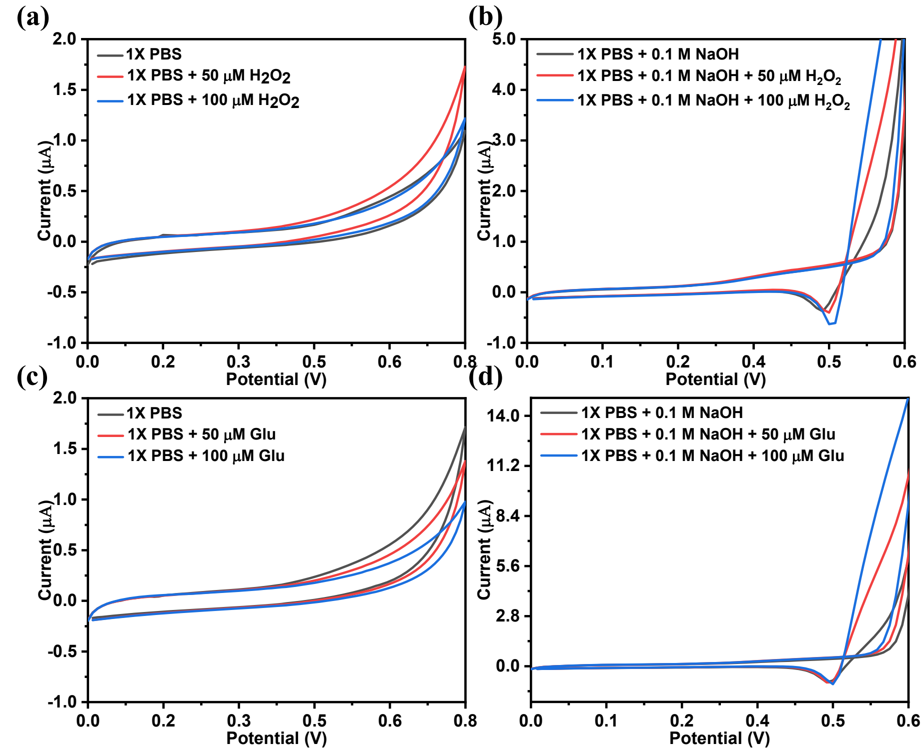

Figure S5. (**a**) CV of Ni-HHTP/SPCE in 1X PBS, 1X PBS containing 50 μM H_2O_2, and 1X PBS containing 100 μM H_2O_2. (**b**) CV of Ni-HHTP/SPCE in 1X PBS containing 0.1 M NaOH, 1X PBS containing 0.1 M NaOH and 50 μM H_2O_2, 1X PBS containing 0.1 M NaOH and 100 μM H_2O_2. (**c**) CV of Ni-HHTP/SPCE in 1X PBS, 1X PBS containing 50 μM Glu, and 1X PBS containing 100 μM Glu. (**d**) CV of Ni-HHTP/SPCE in 1X PBS containing 0.1 M NaOH, 1X PBS containing 0.1 M NaOH and 50 μM Glu, 1X PBS containing 0.1 M NaOH and 100 μM Glu.

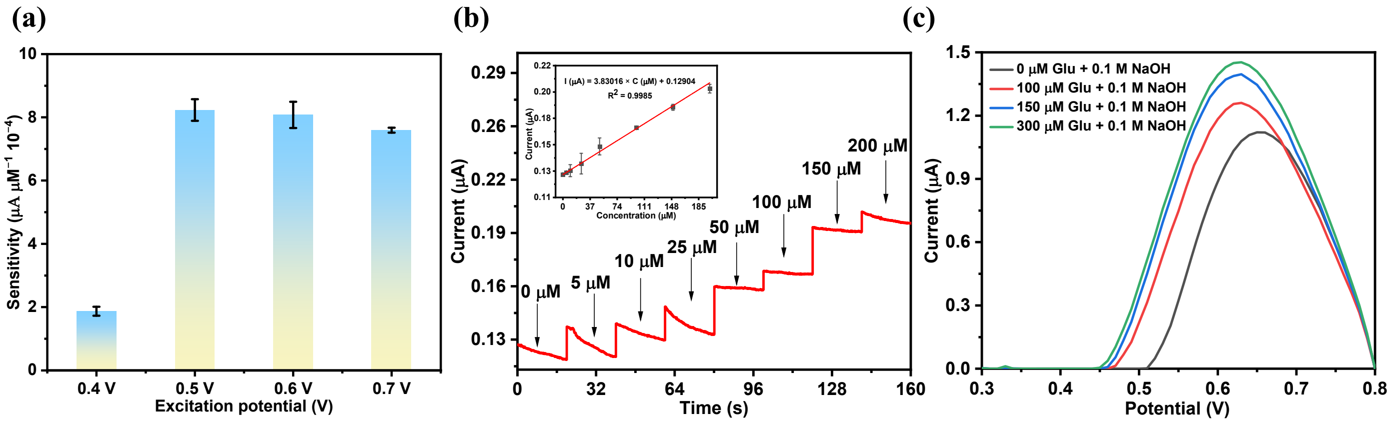

Figure S6. (a) The slope of Ni-HHTP/SPCE curve fitted by CA at the excitation potential of 0.4V, 0.5V, 0.6V and 0.7V. (0.1M NaOH containing 50 μM Glu, 150 μM Glu and 300 μM Glu). **(b)** CA of Ni-HHTP/SPCE at 0.5 V excitation potential for detecting different concentrations of Glu (inset shows the corresponding calibration curve of current versus Glu concentrations ranging from 0 to 200 μM). **(c)** DPV of Ni-HHTP/SPCE in 0.1 M NaOH containing 0 μM Glu, 100 μM Glu, 150 μM Glu, 300 μM Glu , respectively.

The limit of detection (LOD) is the lowest concentration or content of the analyte that can be detected, and the smaller the LOD, the higher the sensitivity of the detector [66]. The calculation formula is:

$$LOD = 3 \times SD / S \quad (S1)$$

Where S is the slope of the calibration curve and SD is the standard deviation of the blank parallel samples. We tested five sets of blank parallel samples for CA and then listed the response currents in Table S1 and calculated the SD to be 0.0050. In addition, the S of the standard curve in Figure 4b is 0.0070534. Therefore, the LOD of the biosensor for H_2O_2 concentration is calculated as 2.1266 μM. Similarly, the LOD of the biosensor for Glu concentration is calculated as 1.2963 μM.

Table S1. Detection of blank sample (H_2O_2 sensor).

blank sample	1	2	3	4	5
**Response current (μA)**	0.443	0.441	0.439	0.450	0.437
**Standard deviation (SD)**			0.0050		

Table S2. Detection of blank sample (Glucose sensor).

blank sample	1	2	3	4	5
**Response current (μA)**	0.253	0.238	0.187	0.246	0.256
**Standard deviation (SD)**			0.0944		

Table S3. Comparison of MOFs based nonenzymatic glucose sensors.

Electrode Material	Substrate	Sensitivity ($\mu A\ mM^{-1}\ cm^{-2}$)	LOD (μM)	Sample	Reference
Ni@Cu-MOF	GCE	1703.33	1.67	human serum	67
Ni-MOF	/	21744	0.66	human serum	68
Ni–Co MOF/Ag/rGO	PU	425.9	3.28	sweat	34
Ni–Co MOF/Au	PDMS	205.1	4.25	sweat	68
MOF-74(Cu) NS-CC	GCE	3350	0.41	human serum	70
Ni-MOF/CNTs	GCE	13850	0.82	human serum	63
Ni-HHTP	SPCE	5573.21	1.30	sweat	This work

Table S4. Comparison of different nonenzymatic electrochemical H_2O_2 sensors.

Electrode Material	Substrate	Sensitivity ($\mu A\ mM^{-1}\ cm^{-2}$)	LOD (μM)	Reference
Co-MOF	GCE	83.10	3.76	71
Pt NPs@UiO-66-2	GCE	75.33	3.06	72
NiMn-LDH/GO	GCE	96.82	4.40	73
2-bromo-3-ferrocenyl-1,4-naphthoquinone	GCE	71.40	2.7	74
Ni-HHTP	SPCE	179.85	2.13	This work
